# G2 Strain of Rotavirus among Infants and Children, Bangladesh

**DOI:** 10.3201/eid1501.080883

**Published:** 2009-01

**Authors:** Shuvra Kanti Dey, Yuko Hayakawa, Majibur Rahman, Rafiqul Islam, Masashi Mizuguchi, Shoko Okitsu, Hiroshi Ushijima

**Affiliations:** The University of Tokyo, Tokyo, Japan (S.K. Dey, M. Mizuguchi, H. Ushijima); Society for Rural Education and Development, Dhaka, Bangladesh (R. Islam); University of Dhaka, Dhaka (M. Rahman); Gunma Paz College, Gunma, Japan (Y. Hayakawa); Aino College, Tokyo (S. Okitsu).

**Keywords:** Rotavirus, RT-PCR, G-type, P-type, emergence, dispatch

## Abstract

To determine G and P genotypes, we performed nested PCR on 307 rotavirus specimens collected in Dhaka, Bangladesh, during 2004–2005. G2 (43.3%) was detected at the highest frequency, followed by G4 (19.5%), G9 (13.7%), G1 (12.7%), and G3 (2.6%). P[8] was the most predominant genotype (53.2%), followed by P[4] (42.9%).

Group A rotavirus (RAV) is the leading cause of severe gastroenteritis in infants and young children worldwide and accounts for ≈600,000 deaths in children <5 years of age (*1*). Rotaviruses are members of the *Reoviridae* family (*2*) and are classified into 7 groups (A–G) on the basis of distinct antigenic and genetic properties (*3*). On the basis of neutralization assay and sequence analysis, a total of 15 G and 27 P genotypes of RAV have been documented (*4*). The major human G types are G1, G2, G3, G4, and G9, which, when combined with the P types P[8], P[4], and P[6], account for >80% of rotavirus-associated gastroenteritis (*5*).

## The Study

A total of 917 stool specimens were collected from infants and children with acute gastroenteritis in Dhaka Children’s Hospital, Bangladesh, during October 2004 to September 2005. Fecal specimens were diluted with distilled water to 10% suspensions and clarified by centrifugation at 10,000 × *g* for 10 min. The supernatant was collected and viral genomes were extracted from fecal specimens by using the QIAamp viral RNA Mini Kit (QIAGEN, Hilden, Germany). Using reverse transcription–PCR (RT-PCR) with specific primers, as previously reported (*6*), resulted in the identification of diarrheal viruses, including group A, B, and C rotaviruses and adenovirus.

RAV-positive samples were then subjected to G and P genotyping by nested PCR with previously published primers (*7*–*9*).The RAV isolate for which the G and P types could not be determined by RT-PCR method was subjected to nucleotide sequence analysis of PCR products positive for VP7 and VP4 genes with the BigDye Terminator Cycle Sequencing Kit and ABI Prism 3100 Genetic Analyzer (Applied Biosystems Inc., Foster City, CA, USA). Their VP7 and VP4 nucleotide sequences were compared as well as those of reference rotavirus strains available in GenBank by using BLAST (http://blast.ncbi.nlm.nih,gov/Blast.cgi). Phylogenetic and molecular evolutionary analyses were conducted by using MEGA version 3.2 software (*10*). The sequences of VP4 and VP7 genes of rotaviruses detected in this study had been submitted to GenBank under accession nos. EU855813–EU855822 and EU855823–EU855830, respectively.

Among diarrhea-causing viruses detected, RAV was the most prevalent (33.5%), followed by adenoviruses (1.9%). Rotavirus group B and rotavirus group C could not be detected in this study. Ninety-seven percent of RAV-infected patients were hospitalized. Co-infection between RAV and adenoviruses was identified in 7 of 917 samples. Most of the patients in this study were 1 to 24 months of age; RAV infection was most commonly detected in patients 6 to 23 months of age. Gender distribution of patients with RAV-positive samples was 56% male and 44% female.

**Figure 1:**
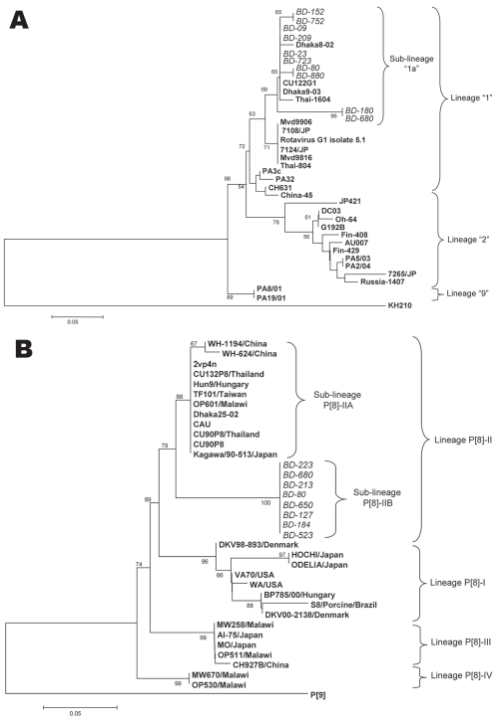
Phylogenetic analysis of the nucleotide sequences of the VP7 and VP4 genes of untypeable group A rotavirus strains (RAV) from Bangladesh. A) Neighbor-joining phylogenetic tree based on nucleotide sequences of the VP7 encoding genes for untypeable RAV strains. B) Neighbor-joining phylogenetic tree based on nucleotide sequences of the VP4 encoding genes for untypeable RAV strains. The numbers in the branches indicate the bootstrap values. Reference strains of RAV G1 and P[8] strains were selected from DNA database of Japan/GenBank under the accession number indicated in **boldface**. G1 strains from Bangladesh are highlighted in *italics*. Scale bars indicate nucleotide substitutions per position. Reference RAV strains used in this study and their accession numbers are as follows: RAV P[8] strains: BP785/00/Hungary (AJ605315), VA70/USA (AJ540229), WA/ USA (L34161), HOCHI/Japan (AB039943), ODELIA/Japan (AB039942), MW670/Malawi (AJ302146), OP530/Malawi (AJ302152), AI-75/Japan (AB008285), MW258/Malawi (AJ302143), OP511/Malawi (AJ302151), CH927B/China (AB008273), MO/Japan (AB008278), Kagawa/90-513/Japan (AB039944), OP601/Malawi (AJ302153), CU132P8/Thailand (DQ235955), DK V98-893/Denmark (AY509908), DK V00- 2138/Denmark (AY509910), S8/Porcine/Brazil (AF052449), CU90P8/Thailand (DQ235978), TF101/Taiwan (AF183870), Hun9/Hungary (AJ605320), WH-1194/China (AY856445), Dhaka25-02 (DQ146652), CU90P8 (DQ235978), 2vp4n (DQ675009), CAU 164 (EU679398) and WH-624/China (AY856444); RAV G1 strains: Dhaka9-03 (DQ482715), CU122G1 (DQ236053), PA5/03 (DQ377596), KH210 (AB303218), 7014/JP (EF079064), rotavirus G1 isolate 5.1(DQ672628), Mvd9906 (AF480278), 7265/JP (EF079066), 7124/JP(EF079069), 7108/JP (EF079068), JP421 (D16326), Fin-408 (Z80303), PA2/04 (DQ377598), Fin-429 (Z80312), AU007 (AB081799), G192B (AF043678), DC03 (AF183859), Oh-64 (U26387), PA3c (DQ377566), PA32 (DQ377574), Thai-1604 (DQ512981), Dhaka8-02 (AY631049), Thai-804 (DQ512979), Mvd9816 (AF480293) CH631 (AF183857), China-45 (U26371), Russia-1407 (S83903), PA8/01 (DQ377592), PA19/01 (DQ377593).

We could not initially determine G type for 10 RAV and P type for 8 RAV isolates, even though their VP7 and VP4 genes were successfully amplified by RT-PCR. Sequence analysis showed that all of untypeable RAV were G1 and P[8]. Ten rotavirus G1 sequences were classified into a distinct lineage, lineage1 and sublineage 1a. G1 strains analyzed in this study belonged to the Asian cluster and were most closely related to Dhaka-02, Dhaka-03, and Thai-1602 strains, which had high identities at the nucleotide level with each other (99%–100%). Eight rotavirus P[8] sequences in this study belonged to 1 distinct lineage, lineage P[8]-II, but made a novel sublineage, sublineage P[8]-IIB, which had a high nucleotide sequence identity of 100% within lineage P[8]-II (Figure 1). These untypeable rotavirus P[8] strains contained 2–3 point mutations at the VP4 primer-binding site.

**Figure 2:**
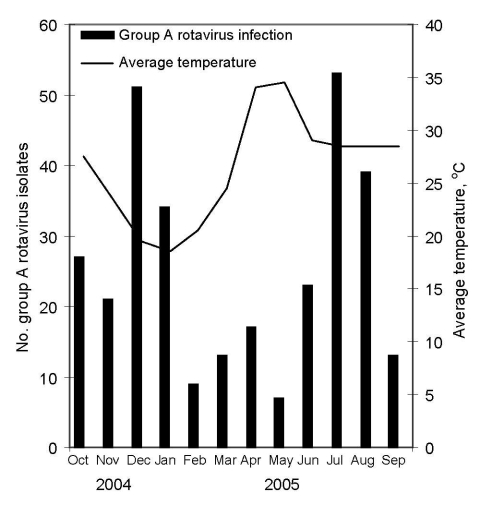
Seasonal pattern of group A rotavirus infection in infants and children with acute gastroenteritis in Dhaka, Bangladesh, October 2004–September 2005.

RAV was detected all year round, but 2 peaks in infections occurred: 1 peak apparently lasted 4 months (October 2004 through January 2005) and another peak lasted 3 months (June 2005 through August 2005) (Figure 2). Five different G types, G1–G4 and G9, were detected during the study period. Of these, G2 was the most common (43.3%) followed by G4 (19.5%), G9 (13.7%), G1 (12.7%), and G3 (2.6%). Mixed infections between >1 G genotypes were identified in 4% of the specimens (Table). Genotype G2 was detected in every month with a relatively high incidence rate. Among 307 RAV-positive samples, 280 samples were P typed successfully, and P[8] was the most predominant genotype (53.2%), followed by P[4] (42.9%) and mixed infections between different P genotypes (5.0%). G2P[4] combination was the most predominant genotype (39%), followed by G4P[8] (18.2%), G9P[8] (13%), G1P[8] (11.8%), and G3P[8] (2.9%).

**Table:** Distribution of group A rotavirus G and P genotypes among infants and children with acute gastroenteritis in Dhaka City, Bangladesh, 2004–2005*

Genotype	P[8]	P[4]	P[6]	Mixed*	Nontypeable	Total
G1	33	4	0	1	2	40
G2	11	109	0	9	13	142
G3	8	0	0	1	0	9
G4	51	4	0	2	5	62
G9	36	2	1	2	3	44
Mixed*	10	0	0	0	0	10
Total	149	119	1	15	23	307

*>1 G or P genotype was recognized.

## Conclusions

Of 917 fecal specimens tested, 307 (33.5%) were positive for RAV. This result was consistent with the previous findings on rotavirus epidemiology in Bangladesh in which its prevalence was ≈29% (*11*,*12*). Our study demonstrated 2 peaks of rotavirus infection. The winter rotavirus peak is usually observed worldwide, but the monsoon peak is not common in settings with temperate climates. Why there was a relation between rainy season and viral infection in this study is not clear. We identified most of the globally common rotavirus types (G1, G2, G4, and G9) in our study. Even though G3 is one of the most prevalent rotavirus types worldwide, the G3 strain has not been detected in Bangladesh since 1993 (*13*). However, we found that 4% of the rotavirus types identified in this study were G3. Results of rotavirus diversity from this study were compared with results of previous studies in Bangladesh (*13*), and we found that G2 was a predominant rotavirus strain among infants and children in Dhaka, Bangladesh.

Rotavirus G4 genotype was the most common genotype in Dhaka from 1992 through 1997 but became a less common rotavirus strain over time; G9 was the leading genotype followed by G2, G4, and G1 in Dhaka (*12*,*14*). The prevalence of G9 strains was nearly the same in our study, but G2 strains showed a dramatic increase. From 2001–2004, the most common rotavirus genotype was P[8] (76%); non-P[8] strains constituted ≈20%. We also found that rotavirus P[8] (53.2%) strain was the most prevalent. We found that the 4 most common strains globally, G1P[8], G2P[4], G3P[8], and G4[8], were found in 83.9% of cases. The G1P[8] strains, less common in 2001, became the predominant strains in the following years, but decreased again in 2005–06. Rotaviruses show great genomic diversity, and several studies in different regions of Bangladesh have identified types not targeted by candidate rotavirus vaccines (*11*,*14*). The frequent genomic reassortment among different rotavirus types was accelerated by mixed infection and generated huge genomic diversity (*13*).

RAV has been associated with gastroenteritis outbreaks in infants and children <5 years of age. However, less is known of the age distribution of rotavirus infection in Bangladesh. In this study, infections were most commonly detected in children <2 years of age.

Common clinical symptoms of RAV-infected patients were dehydration (84%), vomiting (69%), abdominal pain (52%), and fever (31%), which are in agreement with previous published reports (*15*). Number of loose stools per day was increased, with most patients (76%) having loose stools 3–5 times per day. Our study is limited because we could not conduct other tests such as enzyme immunoassay or polyacrylamide gel electrophoresis to confirm rotavirus illness. The incidence of rotavirus gastroenteritis identified by RT-PCR could be an overestimate because healthy controls tested by RT-PCR had a 5%–10% general incidence of rotavirus.
